# Neonatal Morbidity and Mortality in Advanced Aged Mothers—Maternal Age Is Not an Independent Risk Factor for Infants Born Very Preterm

**DOI:** 10.3389/fped.2021.747203

**Published:** 2021-11-15

**Authors:** Nasenien Nourkami-Tutdibi, Erol Tutdibi, Theresa Faas, Gudrun Wagenpfeil, Elizabeth S. Draper, Samantha Johnson, Marina Cuttini, Rym El Rafei, Anna-Veera Seppänen, Jan Mazela, Rolf Felix Maier, Alexandra Nuytten, Henrique Barros, Carina Rodrigues, Jennifer Zeitlin, Michael Zemlin

**Affiliations:** ^1^Saarland University Medical Center, Hospital for General Pediatrics and Neonatology, Homburg, Germany; ^2^Saarland University Medical Center, Institute of Medical Biometry, Epidemiology and Medical Informatics, Homburg, Germany; ^3^Department of Health Sciences, University of Leicester, Leicester, United Kingdom; ^4^Clinical Care and Management Innovation Research Area, Bambino Gesù Children's Hospital, IRCCS, Rome, Italy; ^5^Université de Paris, CRESS, Obstetrical Perinatal and Pediatric Epidemiology Research Team, EPOPé, INSERM, INRA, Paris, France; ^6^Department of Neonatology and Neonatal Infectious Diseases, Poznan University of Medical Sciences, Poznan, Poland; ^7^Children's Hospital, University Hospital, Philipps University Marburg, Marburg, Germany; ^8^CHU Lille, Department of Neonatology, Lille, France; ^9^EPIUnit-Institute of Public Health, University of Porto, Porto, Portugal

**Keywords:** preterm birth, neonatal mortality, neonatal mobidity, very low birth weight infants, extremely low birth weight infant, advanced maternal age, very advanced maternal age, neonatal outcome

## Abstract

**Background:** As childbearing is postponed in developed countries, maternal age (MA) has increased over decades with an increasing number of pregnancies between age 35–39 and beyond. The aim of the study was to determine the influence of advanced (AMA) and very advanced maternal age (vAMA) on morbidity and mortality of very preterm (VPT) infants.

**Methods:** This was a population-based cohort study including infants from the “Effective Perinatal Intensive Care in Europe” (EPICE) cohort. The EPICE database contains data of 10329 VPT infants of 8,928 mothers, including stillbirths and terminations of pregnancy. Births occurred in 19 regions in 11 European countries. The study included 7,607 live born infants without severe congenital anomalies. The principal exposure variable was MA at delivery. Infants were divided into three groups [reference 18–34 years, AMA 35–39 years and very(v) AMA ≥40 years]. Infant mortality was defined as in-hospital death before discharge home or into long-term pediatric care. The secondary outcome included a composite of mortality and/or any one of the following major neonatal morbidities: (1) moderate-to-severe bronchopulmonary dysplasia; (2) severe brain injury defined as intraventricular hemorrhage and/or cystic periventricular leukomalacia; (3) severe retinopathy of prematurity; and (4) severe necrotizing enterocolitis.

**Results:** There was no significant difference between MA groups regarding the use of surfactant therapy, postnatal corticosteroids, rate of neonatal sepsis or PDA that needed pharmacological or surgical intervention. Infants of AMA/vAMA mothers required significantly less mechanical ventilation during NICU stay than infants born to non-AMA mothers, but there was no significant difference in length of mechanical ventilation and after stratification by gestational age group. Adverse neonatal outcomes in VPT infants born to AMA/vAMA mothers did not differ from infants born to mothers below the age of 35. Maternal age showed no influence on mortality in live-born VPT infants.

**Conclusion:** Although AMA/vAMA mothers encountered greater pregnancy risk, the mortality and morbidity of VPT infants was independent of maternal age.

## Introduction

### Background

The rate of preterm birth has risen in many developed countries during the past decades (1). Recent public health initiatives have led to improved survival rates for very low birth weight (VLBW, <1,500 g) and extremely low birth weight (ELBW, <1,000 g) infants due to progress in both pre- and postnatal care (2, 3). Large multicenter epidemiological research studies have investigated differences between patient cohorts throughout different countries to identify targets for obstetric and/or neonatal interventions (4–8). Randomized controlled trials and technological advances have contributed to this success story, identifying key evidence-based obstetric and neonatal interventions that can be monitored to assess quality of care for very preterm infants (9, 10). Evidence-based practices and the achievement of standardized treatment strategies are main pillars for an improved outcome in preterm infants, especially for those born at the limits of viability (11). The “Effective Perinatal Intensive Care in Europe” (EPICE) study has confirmed the impact of evidence-based practices on improved neonatal outcomes and revealed underuse of evidence-based care in many regions (12).

Social changes in developed countries and the increased availability of assisted reproductive technology (ART) have had a major impact on childbearing patterns (13), and the number of women who delay childbirth until an advanced age of 35 years and above is continuously increasing in most western countries (14). Several studies have explored advanced and very advanced maternal age (AMA/vAMA), in association with pregnancy-related morbidities (15) and mode of obstetric interventions (15–20) and large cohort studies have analyzed data of term newborns (21). However, studies of the outcomes of very preterm infants born to mothers of AMA/vAMA remain sparse (22–28). An important aim of combined obstetric and neonatal care is to reduce morbidities and improve neonatal outcome by addressing risk factors (20, 29). The decision to provide active obstetrical management and neonatal intensive care is complex and requires a team approach with discussions between the obstetric and neonatal teams and the expectant parents (30). Infants born very preterm (VPT) remain at risk of developing a wide array of complications, not only in the neonatal unit, but in the long term (31), which remain inversely related to gestational age (32). These have been shown to be evident in infancy and persist into adulthood in VPT populations (33–35). As mortality rates have fallen, the focus for perinatal interventions is to develop strategies to reduce such long-term morbidity (36). Women with AMA/vAMA at the time of delivery will become a more frequent occurrence and research is needed to advise these mothers and their partners appropriately and inform obstetrics regarding ante-and perinatal management of these women.

### Aim

This study aimed to describe the neonatal outcomes of very preterm neonates born to AMA/vAMA mothers. We aimed to evaluate factors contributing either to an improved, equal and/or unfavorable outcome when compared to VPT infants born to mothers below the age of 35.

## Methods

### Study Population

Our study population was derived from the “Effective Perinatal Intensive Care in Europe” (EPICE) cohort, previously described in detail elsewhere (37). The EPICE cohort recruited 10,329 very preterm births of 8,928 mothers, including terminations of pregnancy and stillbirths from 22+0 to 31+6 weeks of gestation. The births occurred between April 2011 and June 2012 in 19 regions in 11 European countries over a period of 12 months (6 months in France). A standardized questionnaire with common definitions was used for an extensive data collection from health records in all maternity and neonatal units. All infants were followed up until death or discharge from hospital to home or long-term care. Exclusion criteria in the current study were terminations of pregnancy, stillbirths, and births with unknown maternal age or below 18 years. In order to minimize possible bias on morbidity and mortality, we focused on very preterm births without severe congenital anomalies (6). Eligible infants were grouped based on maternal age at delivery into a reference group (18–34 years), an advanced maternal age group (AMA 35–39 years) and a very advanced maternal age group (vAMA ≥40 years).

### Maternal, Obstetrical, and Neonatal Characteristics

Perinatal data were collected on the following maternal and neonatal characteristics: maternal age at delivery, native-born status of mother, pre-gestational maternal morbidity (composite measures of chronic hypertension, diabetes, thrombophilia, asthma, cardiac, renal, endocrine, pulmonary, neurological and mental disorders), parity, type of pregnancy (singleton or multiple), hypertensive disorders of pregnancy (composite measures of gestational hypertension, pre-eclampsia, eclampsia and haemolysis elevated liver enzymes, low platelet count), antepartum hemorrhage >20 weeks, preterm premature rupture of membranes >12 h prior to delivery (PPROM), hospital-level of birth (maternity hospitals with level III NICU, as defined locally vs. other hospitals), delivery by cesarean section (CS), spontaneous onset of preterm labor, antenatal corticosteroids (ANC given at least as one dose before delivery), unexpected birth (defined as delivery at home or on the same day as maternal admission to hospital without *in utero* transfer), gestational age (GA defined as the best obstetric assessment using information on antenatal ultrasounds measures and last menstrual period), infant sex, birth weight, small for gestational age (SGA) defined as a birth weight ≤ 10^th^ percentile of intrauterine references developed for the EPICE cohort (38, 39), Apgar score <4 at 5 min, administration of surfactant, need and length of mechanical ventilation (MV), non-steroidal anti-inflammatory drugs or surgical treated patent ductus arteriosus (treated PDA), confirmed early or late onset sepsis, and country.

### Outcomes

Our primary outcome variable was infant mortality defined as in-hospital death before discharge home or into long-term pediatric care. The secondary outcome included a composite of mortality and/or any one of the following major neonatal morbidities: (1) bronchopulmonary dysplasia (BPD) defined as need for oxygen with fraction of inspired oxygen >21% or any respiratory support dependence (non-invasive or mechanical ventilation) at 36 weeks of postmenstrual age. Infants discharged from the neonatal unit on oxygen or with respiratory support before reaching 36 weeks were classified as having BPD (2). Severe brain injury defined as intraventricular hemorrhage (IVH) ≥ grade III or cystic periventricular leukomalacia (cPVL), (3) severe retinopathy of prematurity (ROP ≥ Stage III or ROP requiring laser or cryotherapy), and (4) severe necrotizing enterocolitis (NEC requiring surgery treatment or peritoneal drainage). In addition, the four major neonatal morbidities were analyzed separately as individual outcomes among survivors.

### Ethical Approval

Appropriate ethical approval was in place for all study regions according to national legislations prior to data collection. The study was also approved by the French Advisory Committee on Use of Health Data in Medical Research (CCTIRS) and the French National Commission for Data Protection and Liberties (CNIL).

### Statistical Analysis

Continuous data is presented as mean ± standard deviation or as median with range as appropriate. Number and percentage were reported for categorical parameters. We compared perinatal characteristics and neonatal outcomes of interest between the three maternal age groups using the analysis of variance (ANOVA) test for continuous variables and the Pearson's chi-squared test for categorical variables. Stepwise multivariate logistic regression models were fitted to estimate the odds ratio (OR) after adjusting for confounding variables based on the literature or statistically significant differences between groups in the univariate analyses. The crude effect of maternal age on neonatal outcome variables was first estimated (baseline model), followed by adjustments for selected variables: In model 1 we included non-modifiable maternal and gestational covariates (maternal morbidity, multiparity, multifetal pregnancy, hypertensive disorders of pregnancy, PPROM, SGA, sex of infant, GA below 28 completed weeks) and in model 2 we added potential variables affected by variations in the prenatal care practices (ANC, delivery in a level III neonatal unit, unexpected birth, spontaneous onset of labor, and CS). Finally, among infants admitted to NICU, the analysis was adjusted in addition for neonatal factors such as transport of the newborn within 48 h after birth (inborn) and use of MV (model 3). Results are given as adjusted OR with 95% confidence intervals (95% CI) for the three maternal age groups, using the group aged 18–34 years as reference. All analyses were performed with IBM® SPSS® Statistics 20. A *p* < 0.05 was considered statistically significant without multiple comparison adjustments.

## Results

**Figure 1:**
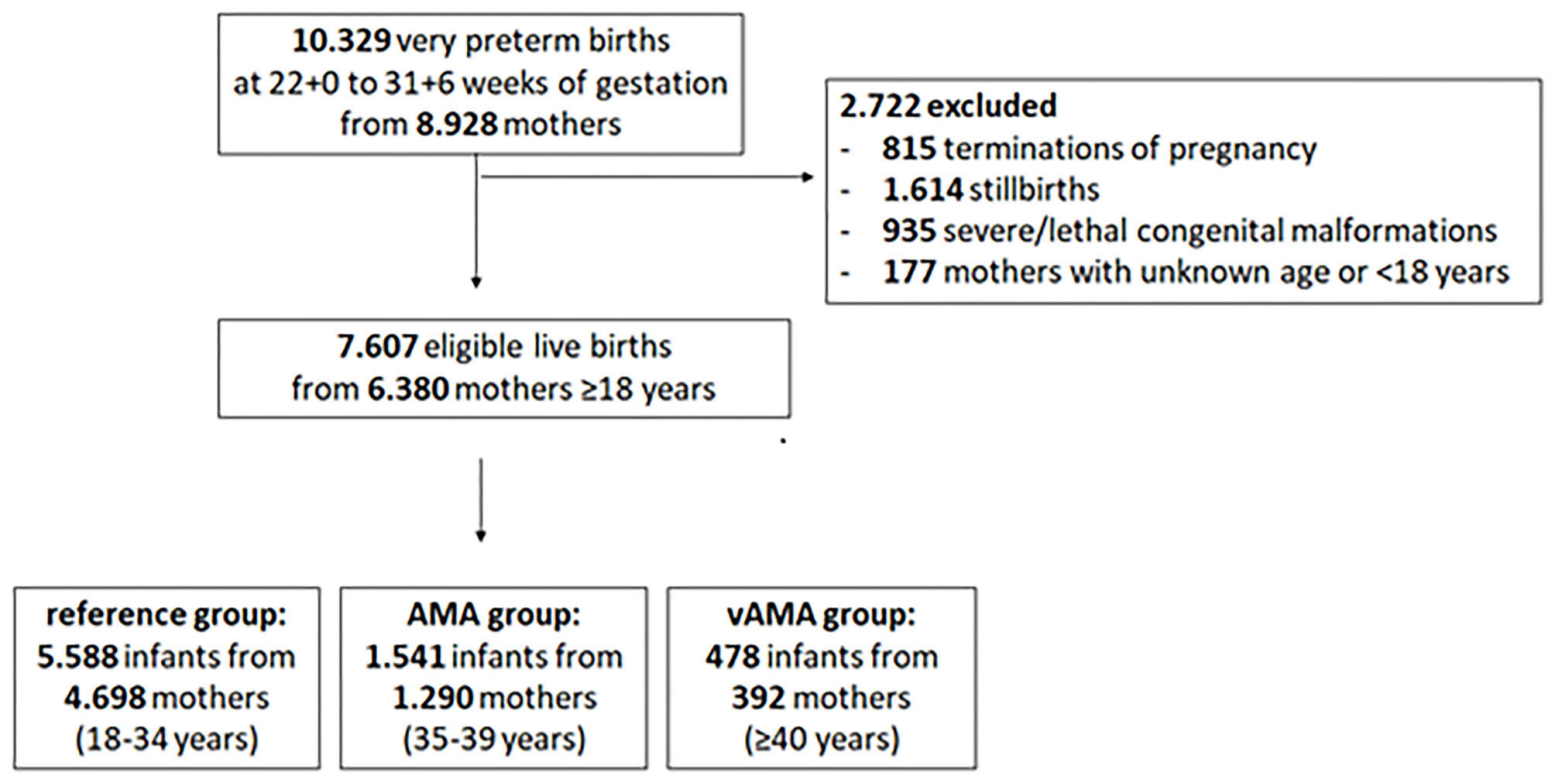
Study population. Flow chart of the EPICE Study population.

The study sample included 7,607 infants born alive to 6,380 mothers (Figure 1), of whom 4.698 (73.6%) were within reference age (27.7 ± 4.3 years), 1.290 (20.2%) had AMA (36.7 ± 1.4 years) and 392 (6.1%) vAMA (41.9 ± 2.4 years).

### Characteristics of Mothers and Pregnancy

Table 1 shows the characteristics of 6.380 mothers and pregnancies according to maternal age. The association tests demonstrated higher rates of multiparity and history of medical problems in both AMA and vAMA mothers compared to younger mothers. In contrast, the rate of native-born mothers and multifetal pregnancies were similar in the three maternal age groups. AMA and vAMA women had higher rates of hypertensive disorders compared to mothers in the reference group. Other complications regarding bleeding, infection and PPROM were not related to mother's age. Obstetric management and interventions showed substantial differences across maternal age groups. Women with AMA and vAMA were significantly less likely have a spontaneous onset of preterm labor or an unexpected delivery on the same day of admission to hospital than the reference group. AMA and vAMA women were most likely to deliver in a level III maternal unit and with cesarean section. Furthermore, mothers with vAMA had significantly higher rate of antenatal corticosteroid administration compared to younger mothers. The average duration of pregnancy was 28.8 ± 2.4 weeks and did not differ between maternal age groups.

**Table 1:** Maternal and pregnancy characteristics of mothers.

	**Missing %**	**Reference (18–34 years)**	**AMA (35–39 years)**	**vAMA (>=40 years)**	**Total**	***P* overall**
Number of mothers		4,698	1,290	392	6,380	
Native-born status	23.7%	77.6%	74.6%	74.9%	76.8%	n.s.
Medical problems^a)^		6.1%	9.8%^∧^	10.2%^#^	7.1%	<0.001
**Current pregancy**						
Multiparity	1.1%	40.8%	57.9%^∧^	58.4%^#^	45.3%	<0.001
Multifetal pregnancy		18.0%	18.8%	20.2%	18.3%	n.s.
**Complications**						
Hypertensive pregnancy disorders		18.2%	21.2%^∧^	25.6%^#^	19.3%	<0.001
Antepartum hemorrhage after week 20	3.1%	22.1%	20.1%	20.7%	21.6%	n.s.
PPROM >12 h	2.3%	26.3%	27.5%	21.6%	26.3%	n.s.
Infection, if indication for delivery	6.4%	9.4%	10.5%	10.3%	9.7%	n.s.
**Delivery management**						
Any antenatal corticosteroids	1.0%	85.9%	87.4%	90.0%^#^	86.4%	0.041
Any antenatal magnesium sulfate	7.7%	7.2%	6.3%	7.2%	7.0%	n.s.
Delivery in level III care unit	0.4%	76.7%	81.3%^∧^	82.1%^#^	78.0%	<0.001
Unexpected birth	5.7%	24.7%	21.8%^∧^	19.9%^#^	23.8%	0.022
Spontaneous onset of labor	1.1%	58.2%	50.2%^∧^	44.0%^#^	55.7%	<0.001
Cesarean section	0.8%	61.5%	72.0%^∧^	76.8%^#^	64.6%	<0.001
Duration of pregnancy, weeks		28.8 ± 2.4	28.9 ± 2.3	29.0 ± 2.3	28.8 ± 2.4	n.s.

a) *Composite measures of chronic hypertension, diabetes, thrombophilia, asthma, cardiac, renal, endocrine, pulmonary, neurological, and mental disorders. Data are n (%) or mean ± standard deviation unless otherwise specified. P were derived from Pearson's Chi-square test or from ANOVA. Mothers with missing data were excluded in the Chi-square test*.

∧ *significant difference AMA vs. reference*,

# *significant difference vAMA vs. reference, n.s. not significant*.

### Characteristics of Infants

In total, 7.607 infants were born alive without severe congenital anomalies to mothers aged ≥18 years. Almost all characteristics of infants, including infant's sex, mean birth weight, extremely low birth weight <1,000 g, mean umbilical pH and Apgar score at 5 min below 4 were not associated with maternal age (Table 2), whereas the proportion of infants being SGA was significantly highest in women with vAMA compared to both mothers with advanced and young age. Infants of vAMA and AMA mothers were significantly more often inborn and had significantly higher mean GA than infants of the reference group. A significantly higher proportion of infants born to vAMA and AMA mother were born with GA at 28–31 weeks as compared to infants from younger mothers. We found no significant association of mean GA and maternal age in any of the GA groups.

**Table 2:** Neonatal characteristics of infants by maternal age group.

	**Missing %**	**Reference (18–34 years)**	**AMA (35–39 years)**	**vAMA (>=40 years)**	**Total**	***P* overall**
Number of infants		5,588	1,541	478	7,607	
Male gender		54.5%	54.2%	49.8%	54.2%	n.s.
Multiple		31.1%	32.0%	34.5%	31.5%	n.s.
Gestational age, weeks		28.8 ± 2.4	29.0 ± 2.3^∧^	29.1 ± 2.3^#^	28.8 ± 2.4	0.007
Gestational age groups						0.05
−22–23 weeks		3.9%	3.4%	2.3%	3.7%	n.s.
−24–27 weeks		28.9%	26.5%	26.2%	28.3%	n.s.
−28–31 weeks		67.1%	70.1%^∧^	71.5%^#^	68.0%	0.02
Birth weight, gram		1190 ± 402	1203 ± 398	1194 ± 389	1194 ± 400	n.s.
Extremely low birth weight <1,000 g		35.2%	32.8%	33.3%	34.6%	n.s.
SGA intrauterine	0.1%	31.2%	32.9%	37.9%^#^^*^	32.0%	0.007
Apgar at 5 min <4	6.2%	6.3%	5.6%	4.5%	6.1%	n.s.
Umbilical cord pH	3.4%	7.29 ± 0.11	7.29 ± 0.11	7.28 ± 0.11	7.29 ± 0.11	n.s.
Death in delivery room		3.3%	2.2%^∧^	2.3%	3.0%	0.022
Death in NICU		9.9%	10.5%	8.4%	10.0%	n.s.
Inborn^a)^)	0.2%	88.4%	90.4%^∧^	91.6%^#^	89.0%	0.02
Surfactant therapy^a)^	2.2%	57.9%	58.8%	52.7%	57.8%	n.s.
Mechanical ventilation^a)^	0.7%	59.9%	57.9%	54.0%^#^	59.1%	0.02
Length of mechanical ventilation, days^a)^	1.0%	6.0 ± 12.6	5.2 ± 11.0	5.7 ± 11.7	5.8 ± 12.1	n.s.
PDA treated^a)^	1.5%	18.8%	19.1%	20.1%	19.0%	n.s.
Postnatal corticosteroids^*a*)^	4.2%	8.8%	7.7%	9.1%	8.6%	n.s.
Sepsis^a)^	1.9%	32.8%	31.2%	33.0%	32.5%	n.s.

*Data are n (%) or mean ± standard deviation unless otherwise specified. P were derived from Pearson's Chi-square test or from ANOVA. Infants with missing data were excluded in the Chi-square test*.

a) *excludes those with death in delivery room (n = 232)*.

∧ *significant difference AMA vs. reference*,

# *significant difference vAMA vs. reference*,

* *significant difference vAMA vs. AMA. n.s. not significant*.

In total, 232 of 7,607 (3.0%) infants died in the delivery room. Delivery room mortality decreased significantly with increasing maternal age group (*p* < 0.022). Half the deaths in the delivery room followed a decision to withhold postnatal resuscitation and decreased significantly in infants of advanced aged mothers—-from 52.6% in reference group to 52.0% in AMA and 50.0% in vAMA (*p* < 0.043). But both delivery room mortality and withhold resuscitation were not associated with maternal age after stratification for gestational age groups. Among the 7.375 infants admitted to NICU, there was no significant difference between the maternal age groups regarding surfactant therapy, use of postnatal corticosteroids to treat BPD, rate of neonatal sepsis and PDA that needed pharmacological or surgical intervention. The infants of vAMA women required significantly less mechanical ventilation during NICU stay than infants of young and AMA mothers, but there was no significant difference for length of mechanical ventilation or after stratification for gestational age groups.

### Neonatal Outcome

Adverse neonatal outcomes did not differ by maternal age group. In the multivariate logistic regression models adjusting for confounding factors, we found no association between maternal age group and the odds of in-hospital mortality, composite outcome of death and/or major neonatal complications, and individual morbidities among the survivors to discharge (Table 3). Unadjusted ORs remained substantially unchanged after adjustment for maternal and gestational (model 1), prenatal care (model 2), and neonatal confounders (model 3).

**Table 3:** Multivariate logistic regression analysis for the association between maternal age and adverse neonatal outcomes in very preterm infants.

		**OR (95% CI)**			
	**Total/No. (%)**	**Crude**	**Adjusted model 1**	**Adjusted model 2**	**Adjusted model 3**
**In-hospital mortality**					
Reference (18–34 years)	5,588/742 (13.3%)	1	1	1	1
AMA (35–39 years)	1,541/196 (12.7%)	1.08 (0.88–1.30)	1.09 (0.88–1.34)	1.10 (0.89–1.36)	1.08 (0.86–1.35)
vAMA (>=40 years)	478/51 (10.7%)	0.83 (0.58–1.18)	0.88 (0.60–1.29)	0.92 (0.63–1.34)	0.95 (0.63–1.43)
**Death and/or any major morbidity**					
Reference (18–34 years)	5,588/1,805 (32.3%)	1	1	1	1
AMA (35–39 years)	1,541/464 (30.1%)	0.92 (0.81–1.04)	1.02 (0.88–1.19)	1.02 (0.88–1.19)	1.02 (0.86–1.20)
vAMA (>=40 years)	478/132 (27.6%)	0.82 (0.66–1.02)	0.96 (0.74–1.24)	0.98 (0.75–1.27)	1.00 (0.76–1.32)
BPD moderate-severe^*^					
Reference (18–34 years)	4,733/772 (16.3%)	1	1	1	1
AMA (35–39 years)	1,317/183 (13.9%)	0.85 (0.71–1.02	0.98 (0.80–1.21)	0.98 (0.80–1.20)	0.97 (0.81–1.17)
vAMA (>=40 years)	422/56 (13.3%)	0.82 (0.61–1.12)	1.05 (0.74–1.47)	1.04 (0.74–1.46)	1.01 (0.75–1.38)
IVH severe or cPVL^*^					
Reference (18–34 years)	4,709/302 (6.4%)	1	1	1	1
AMA (35–39 years)	1,310/81 (6.2%)	0.95 (0.73–1.24)	1.04 (0.79–1.36)	1.05 (0.80–1.38)	0.99 (0.75–1.31)
vAMA (>=40 years)	419/24 (5.7%)	0.97 (0.63–1.49)	1.07 (0.68–1.67)	1.08 (0.69–1.69)	1.12 (0.71–1.76)
ROP severe^*^					
Reference (18–34 years)	4,002/184 (4.6%)	1	1	1	1
AMA (35–39 years)	1,135/40 (3.5%)	0.75 (0.52–1.08)	0.76 (0.51–1.12)	0.77 (0.52–1.14)	0.76 (0.52–1.15)
vAMA (>=40 years)	367/16 (4.4%)	0.79 (0.43–1.42)	0.79 (0.42–1.50)	0.83 (0.44–1.58)	0.81 (0.43–1.55)
NEC severe^*^					
Reference (18–34 years)	4,846/91 (1.9%)	1	1	1	1
AMA (35–39 years)	1,345/22 (1.6%)	0.92 (0.56–1.50)	1.10 (0.66–1.83)	1.10 (0.66–1.83)	1.11 (0.66–1.87)
vAMA (>=40 years)	427/6 (1.4%)	0.58 (0.21–1.58)	0.67 (0.24–1.88)	0.67 (0.24–1.88)	0.65 (0.23–1.84)

*Data are shown as odds ratios with 95% confidence intervals after adjustment for country, pre-gestational maternal morbidity, multiparity, multifetal pregnancy, hypertensive pregnancy disorders, PPROM, SGA, gender of infant, GA below 28 weeks (model 1), + antenatal corticosteroids, delivery in level III, unexpected birth, spontaneous onset of preterm labor, and cesarean section (model 2), + inborn status and use of mechanical ventilation (model 3)*.

* *Among survivors (n = 6,618)*.

## Discussion

### Principal Findings

In line with other large cohort studies, we categorized women into three groups of maternal age. One quarter of infants in this European cohort had mothers with AMA and vAMA. Pregnancy-associated morbidities and co-morbidities of AMA/vAMA in the EPICE patients were comparable to similar patient groups of other large cohort studies (27). We found no differences in duration of pregnancy within the three groups. Multifetal pregnancy was equally distributed among maternal age groups. The incidence of infections was similar throughout the three groups. Except for those VPT born SGA, admitted newborns of AMA/vAMA mothers were equal in gestational age and weight when compared to the reference group of neonates born to mothers under the age of 35. Increasing maternal age was associated with higher rates of hypertensive disorders of pregnancy.

We also found that AMA/vAMA mothers had a significantly higher number of cesarean sections and planned deliveries with very fewer VPT babies born spontaneously, which may contribute to better organized antenatal and postnatal management. The rate of administered antenatal corticoid therapies was significantly higher in the vAMA group. Antenatal corticoid therapy, even when administered shortly before delivery, is known to improve lung function and decrease the need for mechanical ventilation (40). In addition, VPT infants of AMA/vAMA mothers were more often delivered in level III hospitals that provide higher level and more intensive perinatal and postnatal care. The advantage of delivering and/or transferring neonates at high risk to level III centers has been confirmed in recent studies (41, 42). Most importantly, we found no increased mortality in VPT infants of AMA/vAMA mothers in comparison to the reference group of mothers between 18 and 34 years of age. After adjusting for multiple confounders, once admitted to the NICU, VPT infants of AMA/vAMA mothers had the same neonatal outcome when compared with VPT infants of younger mothers. Overall, our data shows that the risks of advanced aged mothers, can be ameliorated at all points of the care pathway. These are major and important findings when it comes to advising these mothers and their partners in antenatal decision making.

### Results in the Context of What Is Known

The improvement in care of infants born VPT remains a major clinical and public health goal, both for obstetricians, and neonatologists. Maternal age and increased pregnancy-associated morbidity with management of the latter are often considered uncertain risk factors when determining neonatal outcome (13, 17, 27). A variety of factors can contribute to adverse pregnancy outcome and/or neonatal outcome. Maternal risk factors such as obesity, gestational diabetes as well other co-morbidities are well-known factors that influence perinatal and neonatal outcomes in term and preterm neonates. Cultural and socio-economic factors play a role when it comes to access, use, and possibility of attendance at antenatal and perinatal care visits (43, 44). Disproportionate use of antenatal care according to risk level of pregnancy indicates the need for better scheduling of care especially in high-risk pregnancies (45, 46). AMA and vAMA are well-known risk factors for adverse pregnancy outcome and pregnancy associated morbidities (18, 19). Many studies have analyzed the impact of aging on obstetric and perinatal outcome among women with AMA/vAMA (19, 21, 27, 47, 48). Yet, data about AMA and outcome of VPT neonates remains sparse (24–26, 28) and contradictory, as some studies report favorable outcomes for VPT infants born to AMA/vAMA mothers (49) and others less favorable outcomes (17). The large amount of heterogeneity among most studies and the neonatal outcomes that were investigated suggest that those results must be interpreted with caution (15, 50). Our study is the first to analyse the effects of AMA/vAMA on the neonatal outcomes for preterm infants in a large cohort with a high number of patients across Europe.

### Clinical Implications

As a vital aim for public child health, we here see an opportunity to re-evaluate national guidelines and build concordant obstetrical and neonatal treatment plans for AMA/vAMA mothers. Regionalization of perinatal care may play a key role and reduction in the deprivation gap remains an important public health goal to secure access to higher level medical care (51–53). Maternal age is one factor that may influence the decision to resuscitate a preterm neonate, especially those at the threshold of viability after birth and increasing maternal age may be positively associated with antepartum and neonatal interventions (54). Once admitted to the NICU, VPT infants of AMA/vAMA mothers do not have higher risks of adverse neonatal outcome as VPT infants—born to mothers below the age of 35. With the idea of shared decision making, advising parents therein, will help guarantee high-level ante-, peri- and postnatal care, crucial for best outcome of their unborn baby. Hence neonatologists and obstetricians should encourage and reinsure parents to exploit all reasonable medical possibilities to influence best outcomes for their child. While counseling AMA/vAMA mothers an emphasis should be given on the importance of level of care especially when facing imminent premature birth.

### Research Implications

Overall, the results of our study raise a few important questions. First, how much perinatal management is necessary regarding current mortality in VPT infants born to AMA/vAMA mothers. Second, which antenatal factors are associated with the good outcome in VPT infant of AMA/vAMA mothers despite their increased risks for complications?

### Strengths and Limitations

The strengths of our study include its prospective design, large sample size and heterogenous population including all level III and non-level-III maternity units in 11 European countries. This facilitates generalizability to a wide range of settings. The availability of data from medical records on all the women who gave birth across 11 European countries within the EPICE study hospitals during the study period abstracted using a standardized form minimizes recall bias. Our study has also limitations regarding the absence of some maternal and neonatal variables such as maternal body mass index, smoking, socioeconomic status, information on delivery room interventions. Some variables are well-known to influence neonatal outcome like maternal obesity for example (55). In this study we did not investigate long term neurological outcomes, yet it is well-known, apart from prematurity, that both intrauterine growth restriction and small-for-gestational age status are associated with impaired childhood cognitive outcomes. As there is a higher number of infants born SGA in AMA/vAMA mothers, long term neurological health problems have to be kept in mind while advising expecting parents (56, 57).

## Conclusion

Improving outcome of VPT infants remains a major global health goal. Due to advances in ART and socio-economical changes in high income countries, motherhood is often postponed to an advanced age. Our study confirms that the growing number of pregnancies beyond the age of 35 or even 40 years have well-known pregnancy complications. Our most important finding is that despite increased pregnancy associated risks and pregnancy complications in AMA/vAMA mothers, VPT infants born to these mothers have the same short-term neonatal outcome as VPT infants born to women below the age of 35 years and do not have a higher risk of mortality and morbidity. These findings are crucial and show that the risks of advanced aged mothers are manageable by modern ante-, peri- and postnatal medicine, indicating that these mothers should be treated at centers that can offer both the best obstetric and neonatal advice and treatment before and after birth to ensure comparable neonatal outcomes for VPT infant born to non-AMA mothers.

## Data Availability Statement

The original contributions presented in the study are included in the article/supplementary material, further inquiries can be directed to the corresponding author/s.

## Ethics Statement

The studies involving human participants were reviewed and approved by national legislations prior to data collection. The European study was also approved by the French Advisory Committee on Use of Health Data in Medical Research (CCTIRS, No. 13.020 on 24/01/2013) and the French National Commission for Data Protection and Liberties (CNIL, No. DR-2013–194 on 10/04/2013 for EPICE). Written informed consent to participate in this study was provided by the participants' legal guardian/next of kin.

## Author Contributions

NN-T, ET, TF, and MZ designed the research and interpreted the results. NN-T, ET, MZ, TF, GW, ED, SJ, MC, RR, A-VS, JM, RM, AN, HB, CR, and JZ collected data and reviewed the data and the manuscript. NN-T, ET, TF, GW, and MZ coordinated data collection and analyzed the data. NN-T wrote the manuscript. All authors contributed to the article and approved the submitted version.

## Funding

The research leading to these results received funding from the European Union's Seventh Framework Programme (FP7/2007-2013 under Grant agreement No. 259882).

## EPICE Research Group

BELGIUM: Flanders (E. Martens, G. Martens, P. Van Reempts); DENMARK: Eastern Region (K. Boerch, A. Hasselager, L. D. Huusom, O. Pryds, T. Weber); ESTONIA (L. Toome, H. Varendi); FRANCE: Burgundy, Ile-de France and Northern Region (P. Y. Ancel, B. Blondel, A. Burguet, P. H. Jarreau, P. Truffert); GERMANY: Hesse (R. F. Maier, B. Misselwitz, S. Schmidt), Saarland (M. Zemlin); ITALY: Emilia Romagna (D. Baronciani, G. Gargano), Lazio (R. Agostino, I. Croci, F. Franco), Marche (V. Carnielli), M. Cuttini, D. DiLallo; NETHERLANDS: Eastern & Central (C. Koopman-Esseboom, A. van Heijst, J. Nijman); POLAND: Wielkopolska (J. Gadzinowski, J. Mazela); PORTUGAL: Lisbon and Tagus Valley (L. M. Graça, M. C. Machado), Northern region (Carina Rodrigues, T. Rodrigues), H. Barros; SWEDEN: Stockholm (A. K. Bonamy, M. Norman, E. Wilson); UK: East Midlands and Yorkshire and Humber (E. Boyle, E. S. Draper, B. N. Manktelow), Northern Region (A. C. Fenton, D. W. A. Milligan); INSERM, Paris (J. Zeitlin, M. Bonet, A. Piedvache).

## Conflict of Interest

The authors declare that the research was conducted in the absence of any commercial or financial relationships that could be construed as a potential conflict of interest.

## Publisher's Note

All claims expressed in this article are solely those of the authors and do not necessarily represent those of their affiliated organizations, or those of the publisher, the editors and the reviewers. Any product that may be evaluated in this article, or claim that may be made by its manufacturer, is not guaranteed or endorsed by the publisher.

## Abbreviations

VLBW: Very low birth weight infants
ELBW: Extremely low birth weight infants
VPT: Infants born very preterm
RCT: Randomized controlled trial
ART: Artificial reproductive medicine
BPD: Bronchopulmonary Dysplasia
CLD: Chronic lung disease
PMA: Postmenstrual age
NICHD: National Institute of Child Health/Human Development
MA: Maternal age
AMA: advanced maternal age
vAMA: very advanced maternal age.

## References

[1] Glass HC Costarino AT Stayer SA Brett CM Cladis F Davis PJ. Outcomes for extremely premature infants. Anesth Analg. (2015) 120:1337–51. 10.1213/ANE.0000000000000705 25988638 PMC4438860

[2] Soll RF. Progress in the care of extremely preterm infants. JAMA - J Am Med Assoc. (2015) 314:1007–8. 10.1001/jama.2015.10911 26348750

[3] Stoll BJ Hansen NI Bell EF Walsh MC Carlo WA Shankaran S. Trends in care practices, morbidity, and mortality of extremely preterm Neonates, 1993-2012. JAMA - J Am Med Assoc. (2015) 314:1039–51. 10.1001/jama.2015.10244 26348753 PMC4787615

[4] Zeitlin J Draper ES Kollée L Milligan D Boerch K Agostino R. Differences in rates and short-term outcome of live births before 32 weeks of gestation in Europe in 2003: Results from the MOSAIC cohort. Pediatrics. (2008) 121:1620. 10.1542/peds.2007-1620 18378548

[5] Draper ES Zeitlin J Fenton AC Weber T Gerrits J Martens G. Investigating the variations in survival rates for very preterm infants in 10 European regions: The MOSAIC birth cohort. Arch Dis Child Fetal Neonatal Ed. (2009) 94:141531. 10.1136/adc.2008.141531 18805823

[6] Draper ES Manktelow BN Cuttini M Maier RF Fenton AC Van Reempts P. Variability in very preterm stillbirth and in-hospital mortality across Europe. Pediatrics. (2017) 139:e20161990. 10.1542/peds.2016-1990 28341800

[7] Smith LK Blondel B Van Reempts P Draper ES Manktelow BN Barros H. Variability in the management and outcomes of extremely preterm births across five European countries: A population-based cohort study. Arch Dis Child Fetal Neonatal Ed. (2017) 102:F400–8. 10.1136/archdischild-2016-312100 28232518

[8] Maier RF Blondel B Piedvache A Misselwitz B Petrou S Van Reempts P. Duration and time trends in hospital stay for very preterm infants differ across European regions^*^. Pediatr Crit Care Med. (2018) 19:1153–61. 10.1097/PCC.0000000000001756 30334907 PMC6282674

[9] Morgan AS Khoshnood B Diguisto C Foix L'Helias L Marchand-Martin L Kaminski M. Intensity of perinatal care for extremely preterm babies and outcomes at a higher gestational age: Evidence from the EPIPAGE-2 cohort study. BMC Pediatr. (2020) 20:8. 10.1186/s12887-019-1856-1 31910799 PMC6945524

[10] Pierrat V Burguet A Marchand-Martin L Cambonie G Coquelin A Roze JC. Variations in patterns of care across neonatal units and their associations with outcomes in very preterm infants: the French EPIPAGE-2 cohort study. BMJ Open. (2020) 10:e035075. 10.1136/bmjopen-2019-035075 32571857 PMC7311036

[11] Brumbaugh JE Hansen NI Bell EF Sridhar A Carlo WA Hintz SR. Outcomes of extremely preterm infants with birth weight less than 400 g. JAMA Pediatr. (2019) 173:434–45. 10.1001/jamapediatrics.2019.0180 30907941 PMC6503635

[12] Zeitlin J Manktelow BN Piedvache A Cuttini M Boyle E Van Heijst A. Use of evidence based practices to improve survival without severe morbidity for very preterm infants: Results from the EPICE population based cohort. BMJ. (2016) 354:i2976. 10.1136/bmj.i2976 27381936 PMC4933797

[13] Sydsjö G Lindell Pettersson M Bladh M Skoog Svanberg A Lampic C Nedstrand E. Evaluation of risk factors' importance on adverse pregnancy and neonatal outcomes in women aged 40 years or older. BMC Pregnancy Childbirth. (2019) 19:1–10. 10.1186/s12884-019-2239-1 30866838 PMC6416921

[14] Sauer M V. Reproduction at an advanced maternal age and maternal health. Fertil Steril. (2015) 103:1136–43. 10.1016/j.fertnstert.2015.03.004 25934599

[15] Leader J Bajwa A Lanes A Hua X Rennicks White R Rybak N. The effect of very advanced maternal age on maternal and neonatal outcomes: a systematic review. J Obstet Gynaecol Canada. (2018) 40:1208–18. 10.1016/j.jogc.2017.10.027 29681506

[16] Delbaere I Verstraelen H Goetgeluk S Martens G De Backer G Temmerman M. Pregnancy outcome in primiparae of advanced maternal age. Eur J Obstet Gynecol Reprod Biol. (2007) 135:41–6. 10.1016/j.ejogrb.2006.10.030 17118520

[17] Yogev Y Melamed N Bardin R Tenenbaum-Gavish K Ben-Shitrit G Ben-Haroush A. Pregnancy outcome at extremely advanced maternal age. Am J Obstet Gynecol. (2010) 203:558.e1–558.e7. 10.1016/j.ajog.2010.07.039 20965486

[18] Klemetti R Gissler M Sainio S Hemminki E. Associations of maternal age with maternity care use and birth outcomes in primiparous women: a comparison of results in 1991 and 2008 in Finland. BJOG An Int J Obstet Gynaecol. (2014) 121:356–62. 10.1111/1471-0528.12415 23944685

[19] Laopaiboon M Lumbiganon P Intarut N Mori R Ganchimeg T Vogel JP. Advanced maternal age and pregnancy outcomes: a multicountry assessment. BJOG. (2014) 121(Suppl. 1):49–56. 10.1111/1471-0528.12659 24641535

[20] Schildberger B Linzner D Hehenberger L Leitner H Pfeifer C. Influence of maternal age on selected obstetric parameters. Geburtshilfe Frauenheilkd. (2019) 79:1208–15. 10.1055/a-0859-0826 31736510 PMC6846731

[21] Molina-García L Hidalgo-Ruiz M Cámara-Jurado AM Fernández-Valero MJ Delgado-Rodríguez M Martínez-Galiano JM. Newborn health indicators associated with maternal age during first pregnancy. Int J Environ Res Public Health. (2019) 16:3448. 10.3390/ijerph16183448 31533243 PMC6765882

[22] Hsieh TT an Liou J Der Hsu JJ Lo LM Chen SF Hung TH. Advanced maternal age and adverse perinatal outcomes in an Asian population. Eur J Obstet Gynecol Reprod Biol. (2010) 148:21–6. 10.1016/j.ejogrb.2009.08.022 19773110

[23] Blomberg M Tyrberg RB Kjølhede P. Impact of maternal age on obstetric and neonatal outcome with emphasis on primiparous adolescents and older women: A Swedish Medical Birth Register Study. BMJ Open. (2014) 4:5840. 10.1136/bmjopen-2014-005840 25387756 PMC4244420

[24] Eventov-Friedman S Zisk-Rony RY Nosko S Bar-Oz B. Maternal age and outcome of preterm infants at discharge from the neonatal intensive care unit. Int J Gynecol Obstet. (2016) 132:196–9. 10.1016/j.ijgo.2015.06.052 26476582

[25] Garite TJ Combs CA Maurel K Das A Huls K Porreco R. A multicenter prospective study of neonatal outcomes at less than 32 weeks associated with indications for maternal admission and delivery. Am J Obstet Gynecol. (2017) 217:72.e1–72.e9. 10.1016/j.ajog.2017.02.043 28267444

[26] Tseng K-T Peng C-C Chang J-H Hsu C-H Lin C-Y Jim W-T. The impact of advanced maternal age on the outcomes of very low birth weight preterm infants. Medicine (Baltimore). (2019) 98:e14336. 10.1097/MD.0000000000014336 30702619 PMC6380823

[27] Kahveci B Melekoglu R Evruke IC Cetin C. The effect of advanced maternal age on perinatal outcomes in nulliparous singleton pregnancies. BMC Pregnancy Childbirth. (2018) 18:343. 10.1186/s12884-018-1984-x 30134873 PMC6106883

[28] Bouzaglou A Aubenas I Abbou H Rouanet S Carbonnel M Pirtea P. Pregnancy at 40 years old and above: obstetrical, fetal, and neonatal outcomes. Is Age An Independent Risk Factor For Those Complications? Front Med. (2020) 7:208. 10.3389/fmed.2020.00208 32537454 PMC7266997

[29] Kollée LAA Cuttini M Delmas D Papiernik E den Ouden AL Agostino R. Obstetric interventions for babies born before 28 weeks of gestation in Europe: Results of the MOSAIC study. BJOG An Int J Obstet Gynaecol. (2009) 116:1481–91. 10.1111/j.1471-0528.2009.02235.x 19583715

[30] Kidszun A Matheisl D Tippmann S Inthorn J Mahmoudpour SH Paul NW. Effect of neonatal outcome estimates on decision-making preferences of mothers facing preterm birth: a randomized clinical trial. JAMA Pediatr. (2020) 174:721–2. 10.1001/jamapediatrics.2020.0235 32310271 PMC7171575

[31] Saigal S Doyle LW. An overview of mortality and sequelae of preterm birth from infancy to adulthood. Lancet. (2008) 371:261–9. 10.1016/S0140-6736(08)60136-1 18207020

[32] Ancel PY Goffinet F Kuhn P Langer B Matis J Hernandorena X. Survival and morbidity of preterm children born at 22 through 34weeks' gestation in france in 2011 results of the EPIPAGE-2 cohort study. JAMA Pediatr. (2015) 169:230–8. 10.1001/jamapediatrics.2014.3351 25621457

[33] Wood NS Marlow N Costeloe K Gibson AT Wilkinson AR. Neurologic and developmental disability after extremely preterm birth. N Engl J Med. (2000) 343:378–84. 10.1056/nejm200008103430601 10933736

[34] Serenius F Källén K Blennow M Ewald U Fellman V Holmström G. Neurodevelopmental outcome in extremely preterm infants at 2.5 years after active perinatal care in Sweden. JAMA - J Am Med Assoc. (2013) 309:1810–20. 10.1001/jama.2013.3786 23632725

[35] Rogers EE Hintz SR. Early neurodevelopmental outcomes of extremely preterm infants. Semin Perinatol. (2016) 40:497–509. 10.1053/j.semperi.2016.09.002 27865437

[36] Ehret DEY Edwards EM Greenberg LT Bernstein IM Buzas JS Soll RF. Association of antenatal steroid exposure with survival among infants receiving postnatal life support at 22 to 25 weeks' gestation. JAMA Netw open. (2018) 1:e183235. 10.1001/jamanetworkopen.2018.3235 30646235 PMC6324435

[37] Zeitlin J Maier RF Cuttini M Aden U Boerch K Gadzinowski J. Cohort profile: Effective perinatal intensive care in Europe (EPICE) very preterm birth cohort. Int J Epidemiol. (2020) 49:372. 10.1093/ije/dyz270 32031620 PMC7266542

[38] Stirnemann JJ Fries N Bessis R Fontanges M Mangione R Salomon LJ. Implementing the INTERGROWTH-21st fetal growth standards in France: a ‘flash study' of the College Français d'Echographie Foetale (CFEF). Ultrasound Obstet Gynecol. (2017) 49:487–92. 10.1002/uog.17223 27516404

[39] Zeitlin J Vayssière C Ego A Goffinet F. More validation is needed before widespread adoption of INTERGROWTH-21st fetal growth reference standards in France. Ultrasound Obstet Gynecol. (2017) 49:547–8. 10.1002/uog.17423 28374438

[40] Norman M Håkansson S Kusuda S Vento M Lehtonen L Reichman B. Neonatal outcomes in very preterm infants with severe congenital heart defects: an international cohort study. J Am Heart Assoc. (2020) 9:e015369. 10.1161/JAHA.119.015369 32079479 PMC7335543

[41] Shah KP Deregnier RAO Grobman WA Bennett AC. Neonatal mortality after interhospital transfer of pregnant women for imminent very preterm birth in Illinois. JAMA Pediatr. (2020) 174:358–65. 10.1001/jamapediatrics.2019.6055 32065614 PMC7042951

[42] Whitham M Dudley DJ. Delivering neonates at high risk in the right place: back to the future again. JAMA Pediatr. (2020) 174:329–30. 10.1001/jamapediatrics.2019.6059 32065605

[43] Gadson A Akpovi E Mehta PK. Exploring the social determinants of racial/ethnic disparities in prenatal care utilization and maternal outcome. Semin Perinatol. (2017) 41:308–17. 10.1053/j.semperi.2017.04.008 28625554

[44] Creanga AA Bateman BT Kuklina E V Callaghan WM. Racial and ethnic disparities in severe maternal morbidity: A multistate analysis, 2008-2010. Am J Obstet Gynecol. (2014) 210:435.e1–435.e8. 10.1016/j.ajog.2013.11.039 24295922

[45] Yeoh PL Hornetz K Dahlui M. Antenatal care utilisation and content between low-risk and high-risk pregnant women. PLoS ONE. (2016) 11:e0152167. 10.1371/journal.pone.0152167 27010482 PMC4807004

[46] Linard M Blondel B Estellat C Deneux-Tharaux C Luton D Oury JF. Association between inadequate antenatal care utilisation and severe perinatal and maternal morbidity: an analysis in the PreCARE cohort. BJOG An Int J Obstet Gynaecol. (2018) 125:587–95. 10.1111/1471-0528.14794 28631308

[47] Zapata-Masias Y Marqueta B Gómez Roig MD Gonzalez-Bosquet E. Obstetric and perinatal outcomes in women ≥ 40 years of age: Associations with fetal growth disorders. Early Hum Dev. (2016) 100:17–20. 10.1016/j.earlhumdev.2016.04.010 27391869

[48] Wu Y Chen Y Shen M Guo Y Wen SW Lanes A. Adverse maternal and neonatal outcomes among singleton pregnancies in women of very advanced maternal age: A retrospective cohort study. BMC Pregnancy Childbirth. (2019) 19:3. 10.1186/s12884-018-2147-9 30606150 PMC6318893

[49] Kanungo J James A McMillan D Lodha A Faucher D Lee SK. Advanced maternal age and the outcomes of preterm neonates: A social paradox? Obstet Gynecol. (2011) 118:872–7. 10.1097/AOG.0b013e31822add60 21934451

[50] Lisonkova S Paré E Joseph KS. Does advanced maternal age confer a survival advantage to infants born at early gestation? BMC Pregnancy Childbirth. (2013) 13:87. 10.1186/1471-2393-13-87 23566294 PMC3637212

[51] Smith LK Budd JLS Field DJ Draper ES. Socioeconomic inequalities in outcome of pregnancy and neonatal mortality associated with congenital anomalies: Population based study. BMJ. (2011) 343:d4306. 10.1136/bmj.d4306 21771825 PMC3139368

[52] Smith LK Manktelow BN Draper ES Springett A Field DJ. Nature of socioeconomic inequalities in neonatal mortality: population based study. BMJ. (2011) 342:38. 10.1136/bmj.c6654 21127118 PMC2996545

[53] Best KE Seaton SE Draper ES Field DJ Kurinczuk JJ Manktelow BN. Assessing the deprivation gap in stillbirths and neonatal deaths by cause of death: A national population-based study. Arch Dis Child Fetal Neonatal Ed. (2019) 104:F624–30. 10.1136/archdischild-2018-316124 30842208

[54] Hajdu SA Rossi RM DeFranco EA. Factors associated with maternal and neonatal interventions at the threshold of viability. Obstet Gynecol. (2020) 135:1398–408. 10.1097/AOG.0000000000003875 32459432

[55] Tanner LD Brock C Chauhan SP. Severity of fetal growth restriction stratified according to maternal obesity. J Matern Neonatal Med. (2020) 1:1–5. 10.1080/14767058.2020.1773427 32482116

[56] Guerby P Bujold E. Early detection and prevention of intrauterine growth restriction and its consequences. JAMA Pediatr. (2020) 174:749–50. 10.1001/jamapediatrics.2020.1106 32453430

[57] Sacchi C Marino C Nosarti C Vieno A Visentin S Simonelli A. Association of intrauterine growth restriction and small for gestational age status with childhood cognitive outcomes: A systematic review and meta-analysis. JAMA Pediatr. (2020) 174:772–81. 10.1001/jamapediatrics.2020.1097 32453414 PMC7251506

